# Supplementary material for: Competition between global and local online social networks
Source: Sci Rep. 2016 Apr 27;6:25116. doi: 10.1038/srep25116 (PMC4846879; doi:10.1038/srep25116)
Supplement: Supplementary Material [file srep25116-s3.pdf]

Supplemental Materials: "Competition between global and local online social networks"

Kaj-Kolja Kleineberg[1,*] and Marián Boguñá[1]
[1]*Departament de Física Fonamental, Universitat de Barcelona, Martí i Franquès 1, 08028 Barcelona, Spain*
(Dated: April 6, 2016)

I. ESTIMATION OF DATA VARIANCE

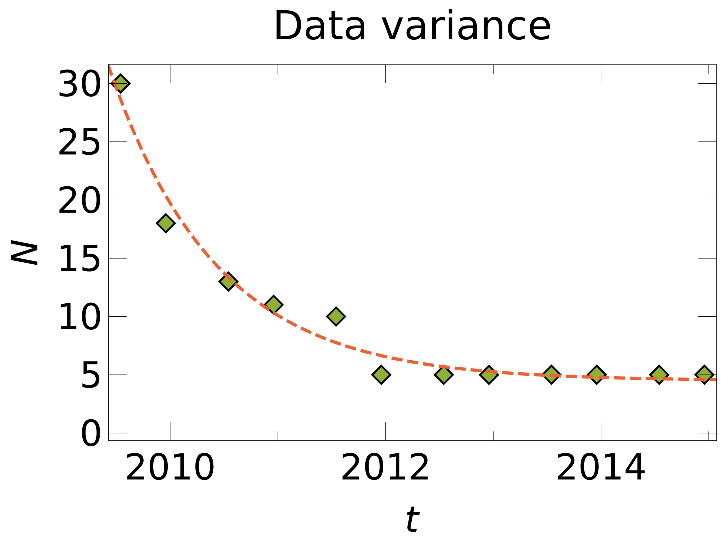

Figure S1: Estimation of data variance. Symbols denote data and the red line a fit (exponential decay).

We estimate the variance σ_N^2 of the data by performing a fit and evaluating the deviation of the datapoints from this fit (see Fig. S1). We find

$$\sigma_N^2 = 1.5\,. \tag{1}$$

II. GOOGLE TRENDS DATA

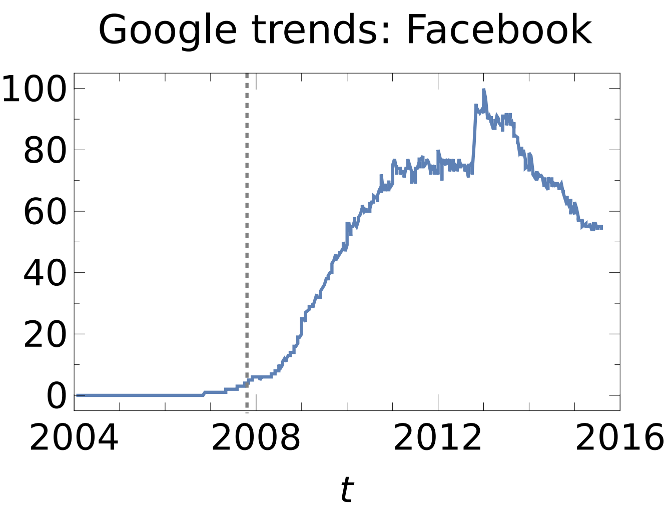

Figure S2: Google trends results for Facebook. The gray dashed line shows the time of global launch predicted by our model.

In Fig. S2 we show the evolution of the search volume for Facebook. The dashed line corresponds to the time of global launch in our model for the best parameter estimate.

*Electronic address: `kkl@ffn.ub.edu`

III. GUIDELINE TO DEVELOP FINE GRAINED DESCRIPTION

In this section, we provide a guideline to generate a fine grained description for the evolution of networks in a certain country or region of interest. Similar to large eddy simulations in fluid dynamics [32,33], we preserve a high accuracy in the region of interest for the prediction and rely on coarse grained approximative dynamics beyond.

The process to create a customized predictive model is as follows.

1. Choose a country of interest.

2. Define the region of influence given by the connections with the highest weight connected to the country of interest.

3. Gather empirical historical data of the evolution of the local networks and the international network in the region of influence.

4. Adjust parameters within the region of interest locally and use generic global parameters for the remaining countries. A suggestion of parameters to adjust locally in the influence region can be found in Tab. I.

Lets consider the example of Brasil (see Fig. S3). A possible choice of the influence region would be the USA, Argentina, Uruguay, Spain, and Portugal. We then propose to adjust the following three parameters locally in the region of influence: the virality λ, the media influence μ, and the launch time delay Δt. Adjusting these parameters in each country would lead to 240 parameters which have to be adjusted simultaneously, which is not feasible and would require much data. However, by performing the above procedure, we reduce significantly the number of parameters (to $\approx 12-18$) and maintain an acceptable level of precision in the region of interest. Hence, this hybrid approach of adjusting parameters locally within the region of influence and globally beyond enables the development of precise customized predictions with a feasible effort.

Quantity	Parameter	Suggestion
Activity affinity	σ	Global
Global connectivity	α	Global
Virality	λ	Local in region of influence
Media influence	μ	Local in region of influence or educated guess [5]
Launch time delay	Δt	Local in region of influence

Table I: Suggestion of parameter adjustment.

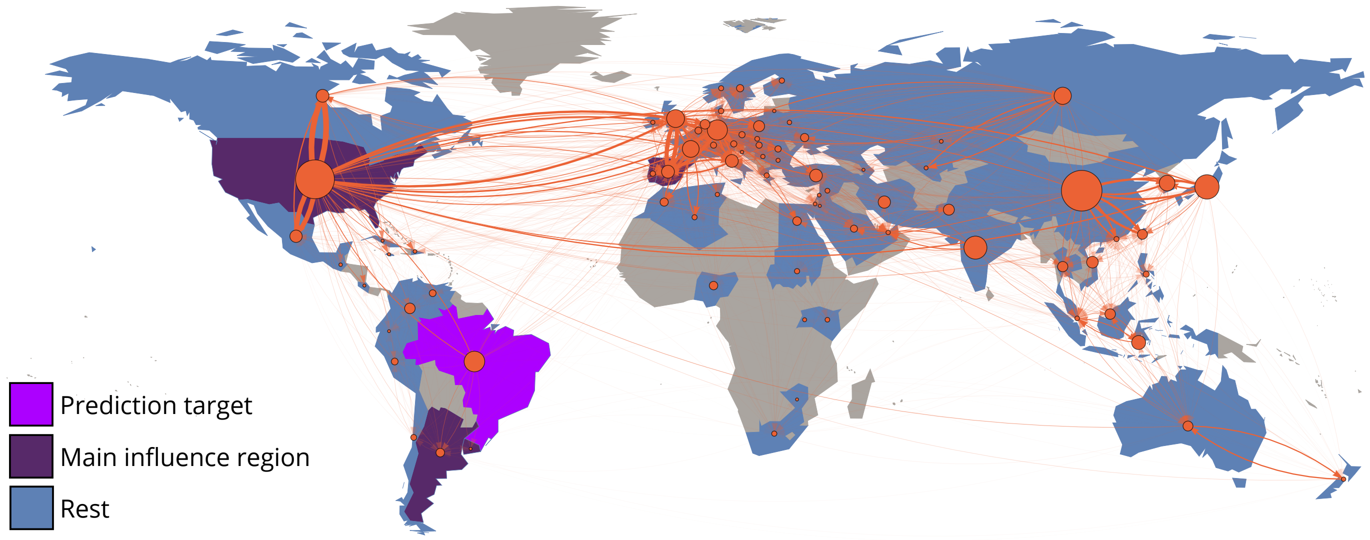

Figure S3: Possible choice of region of influence for Brasil. *Maps created with Mathematica, Version 9, https://www.wolfram.com/mathematica/.*

IV. DOUBLE MEANFIELD APPROXIMATION: $\bar{\Omega} > 0$ BREAKS SYMMETRY OF PITCHFORK BIFURCATION

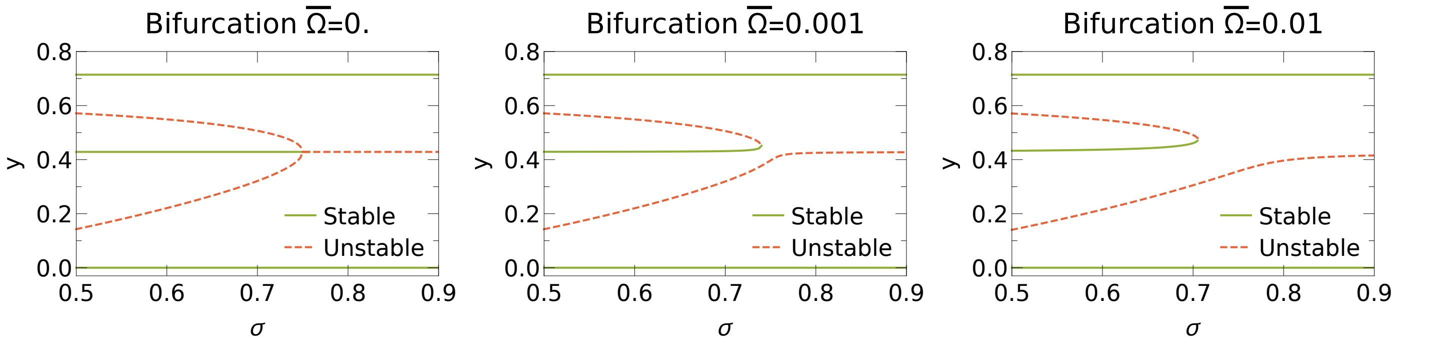

Figure S4: Bifurcation diagram as a function of the control parameter σ for different values of $\bar{\Omega}$. Here, $\lambda \langle k \rangle = 3.5$.

The evolution equations for the double meanfield approximation contain an additional control parameter $\bar{\Omega}$. For $\bar{\Omega} = 0$ we recover the case of two competing identical networks as discussed in [3]. In this case, the system undergoes a subcritical pitchfork bifurcation. Such bifurcation is symmetric locally near the critical point. However, the additional control parameter $\bar{\Omega} > 0$ breaks this symmetry. As a consequence, the system undergoes a saddlenode bifurcation instead of the former pitchfork. See Fig. S4.

V. SUPPLEMENTARY VIDEOS

Video 1: Realization of our model for $\sigma = 0.5$, $\Delta t = 3$, and $\alpha = 1$. Video created with avconv on Ubuntu 14.04 from a sequence of single images created with Mathematica, Version 9, https://www.wolfram.com/mathematica/.

Video 2: Realization of our model for $\sigma = 2$, $\Delta t = 1$, and $\alpha = 1$. Video created with avconv on Ubuntu 14.04 from a sequence of single images created with Mathematica, Version 9, https://www.wolfram.com/mathematica/.

VI. EXPLICIT TIME SERIES

In the following, we present explicit time series of single realizations of our model for different parameters.

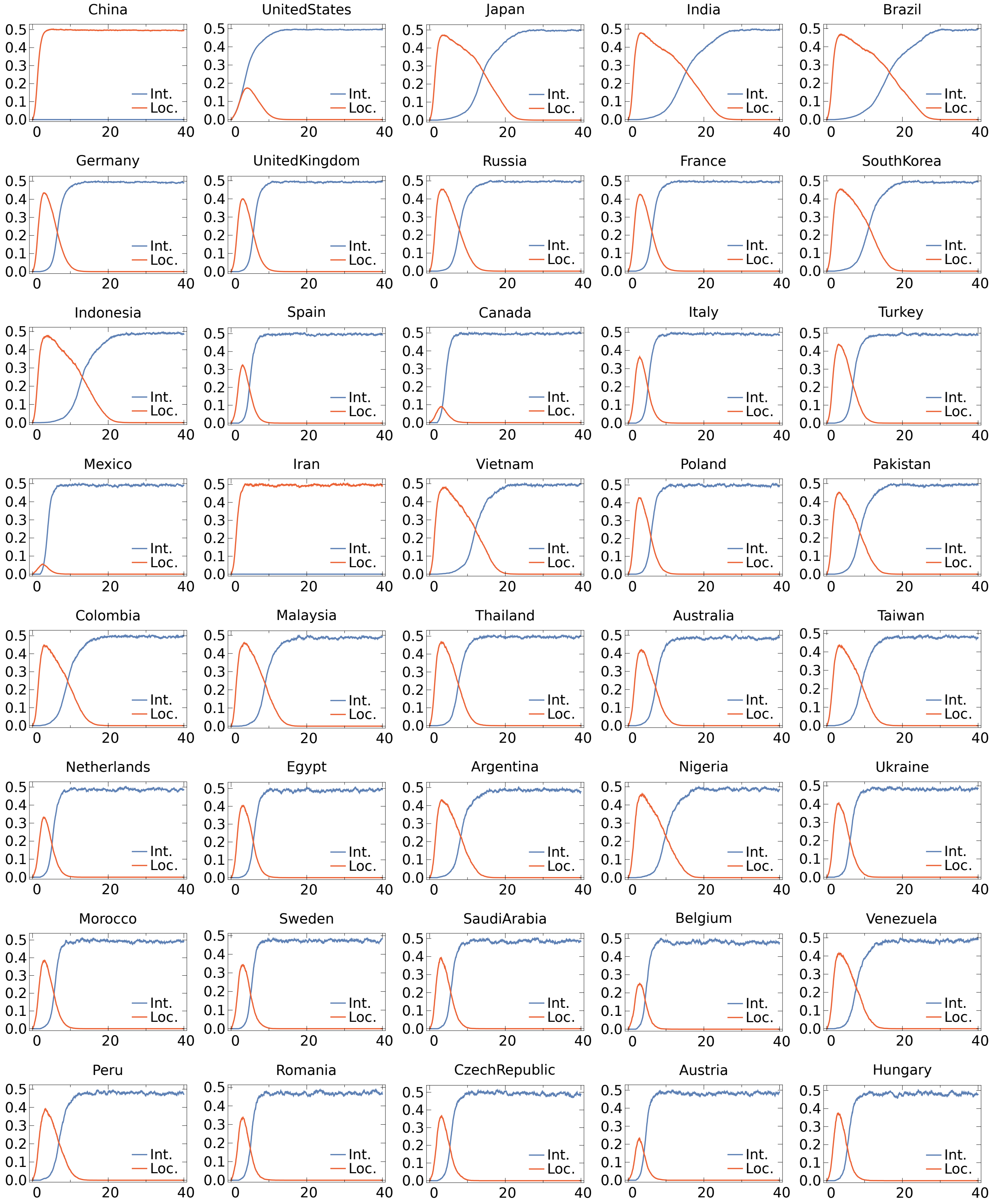

Figure S5: Evolution of network activity for the first 40 countries. Here, $\lambda = 0.2$ per country, $\sigma = 0.75$, $\Delta t = 2$, and $\alpha = 2$.

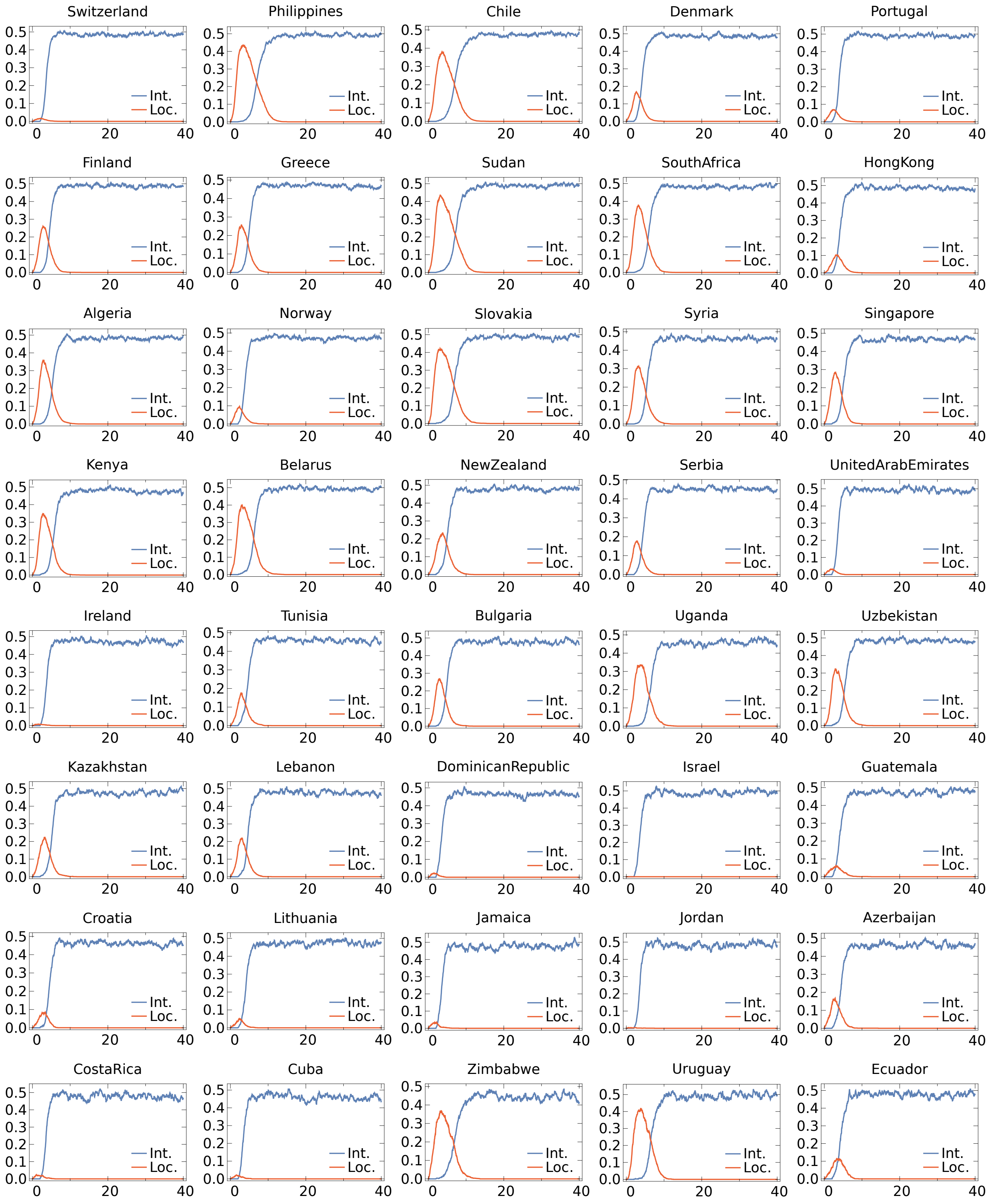

Figure S6: Evolution of network activity for the second 40 countries (continuation from Fig. S5).

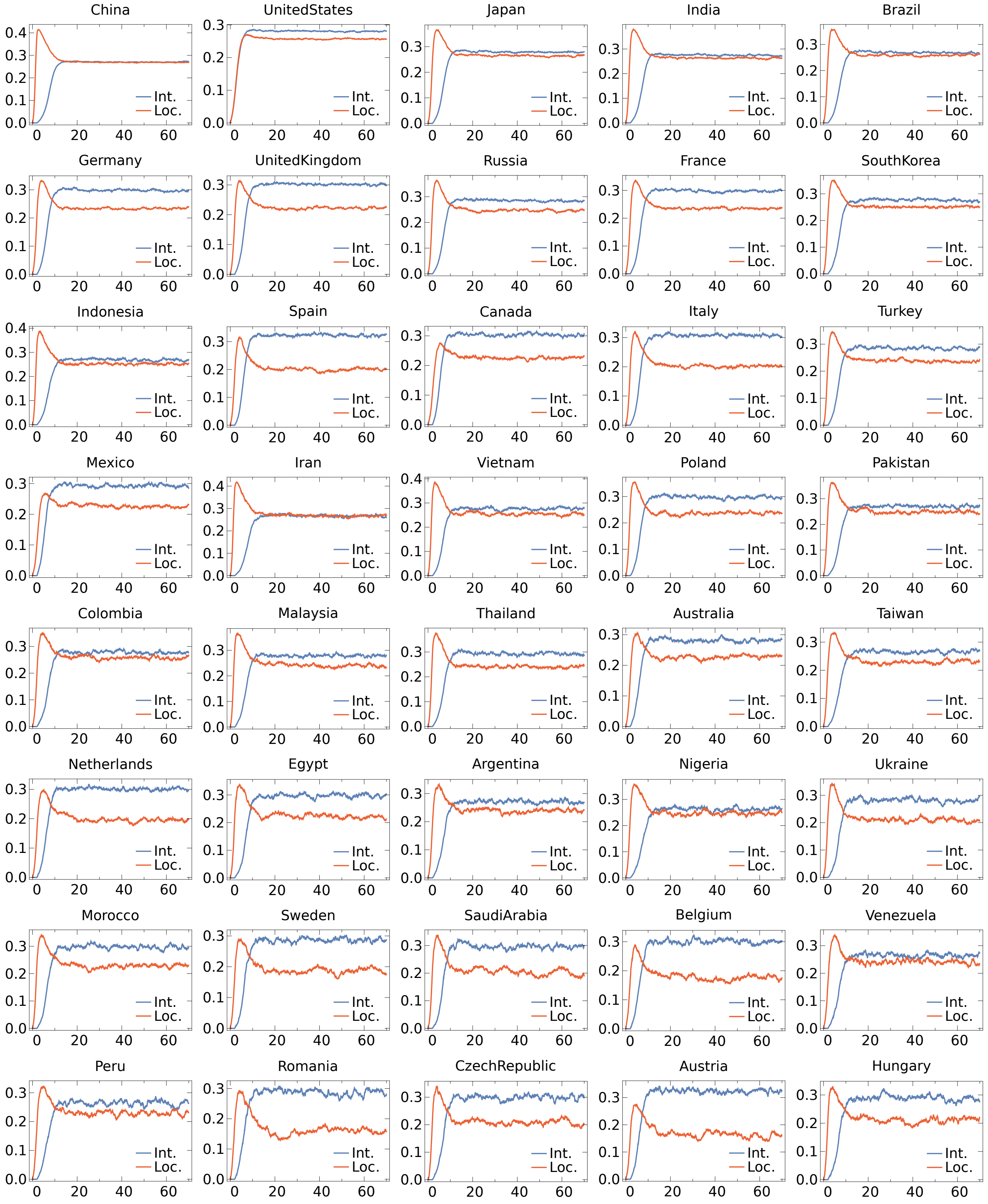

Figure S7: Evolution of network activity for the first 40 countries. Here, $\sigma = 0.25$, $\Delta t = 2$, $\alpha = 0.75$, and $\lambda = 0.2$ per country.

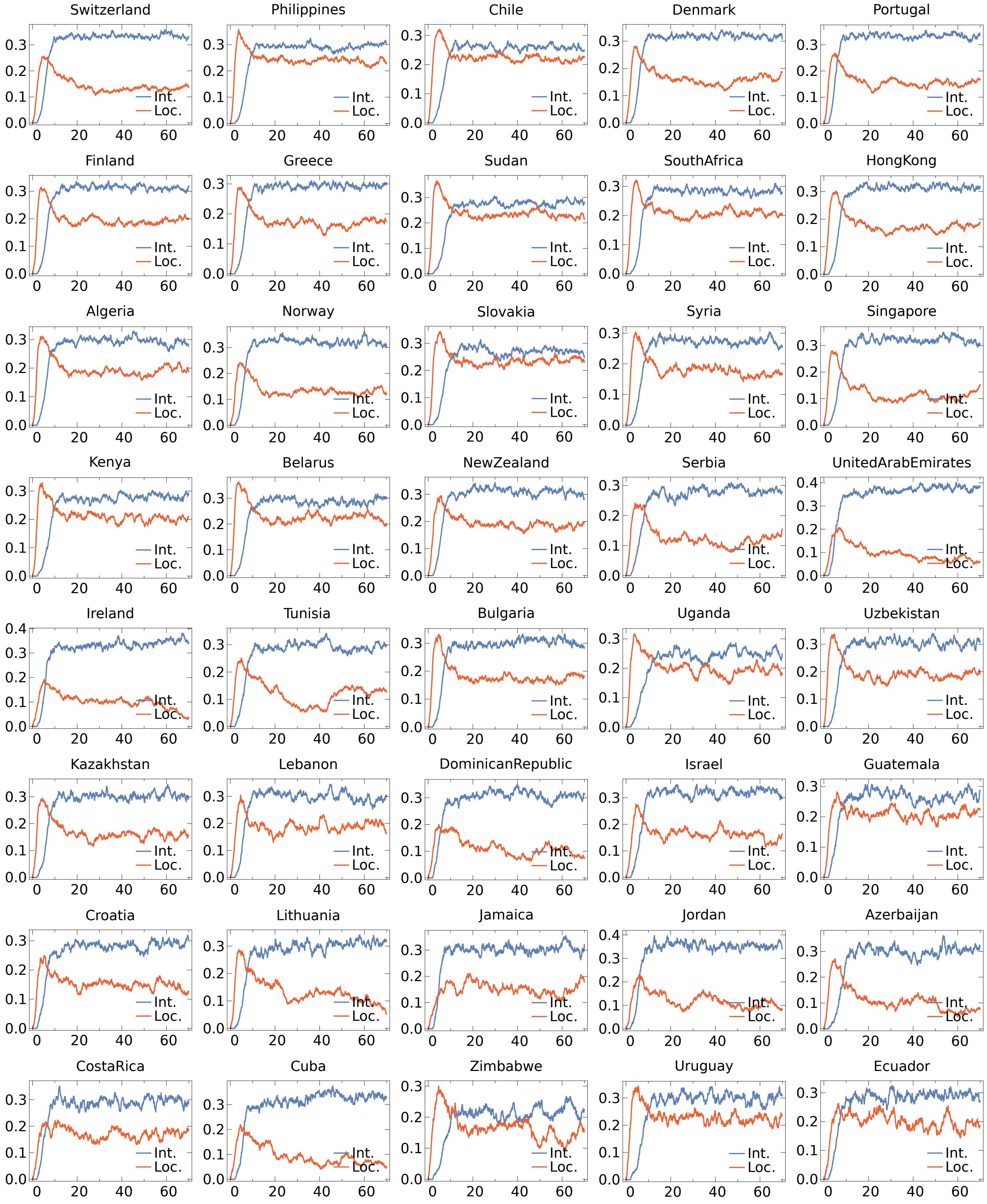

Figure S8: Evolution of network activity for the second 40 countries (continuation from Fig. S7).

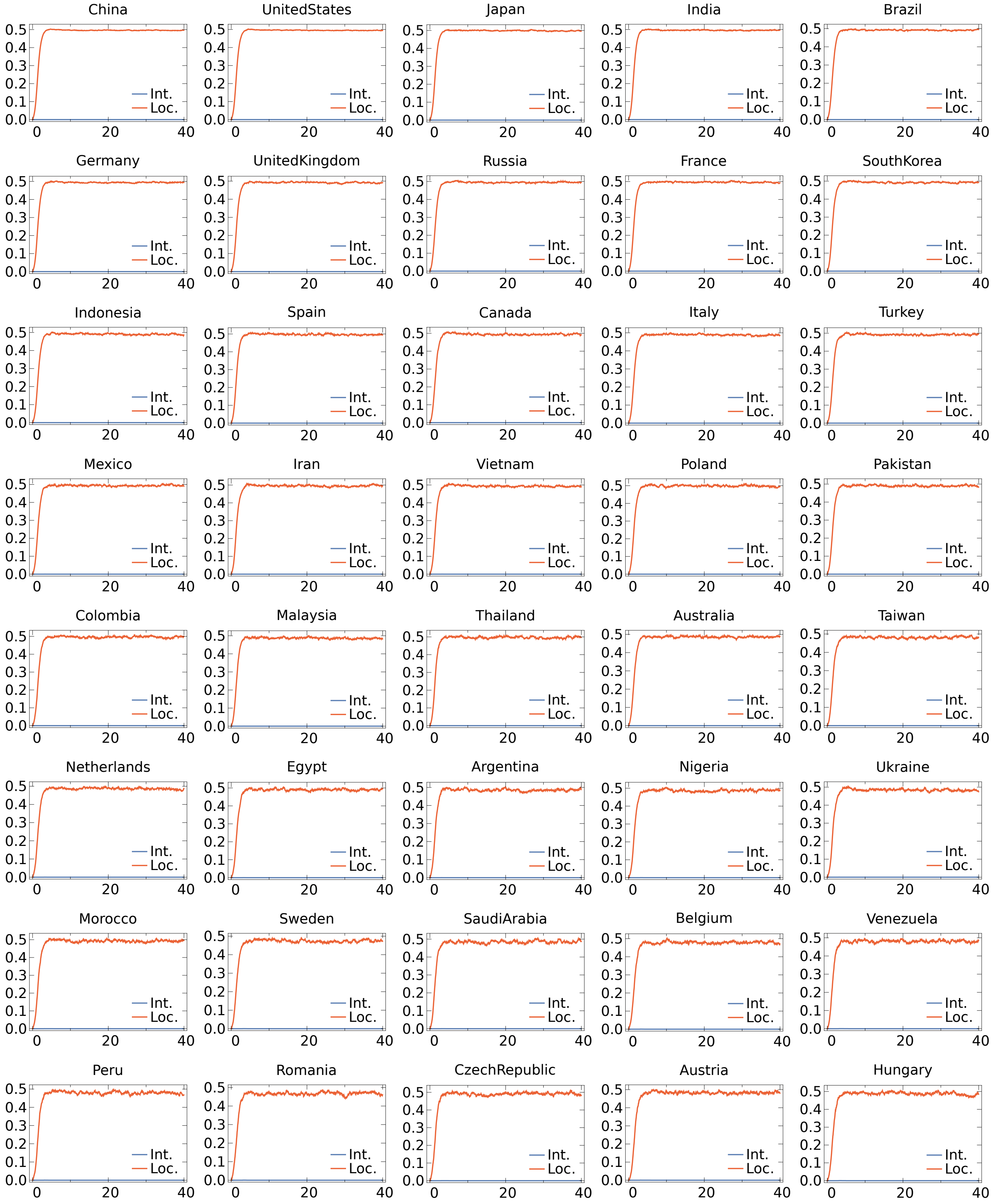

Figure S9: Evolution of network activity for the first 40 countries. Here, $\sigma = 1.5$, $\Delta t = 3$, $\alpha = 2$, and $\lambda = 0.2$ per country.

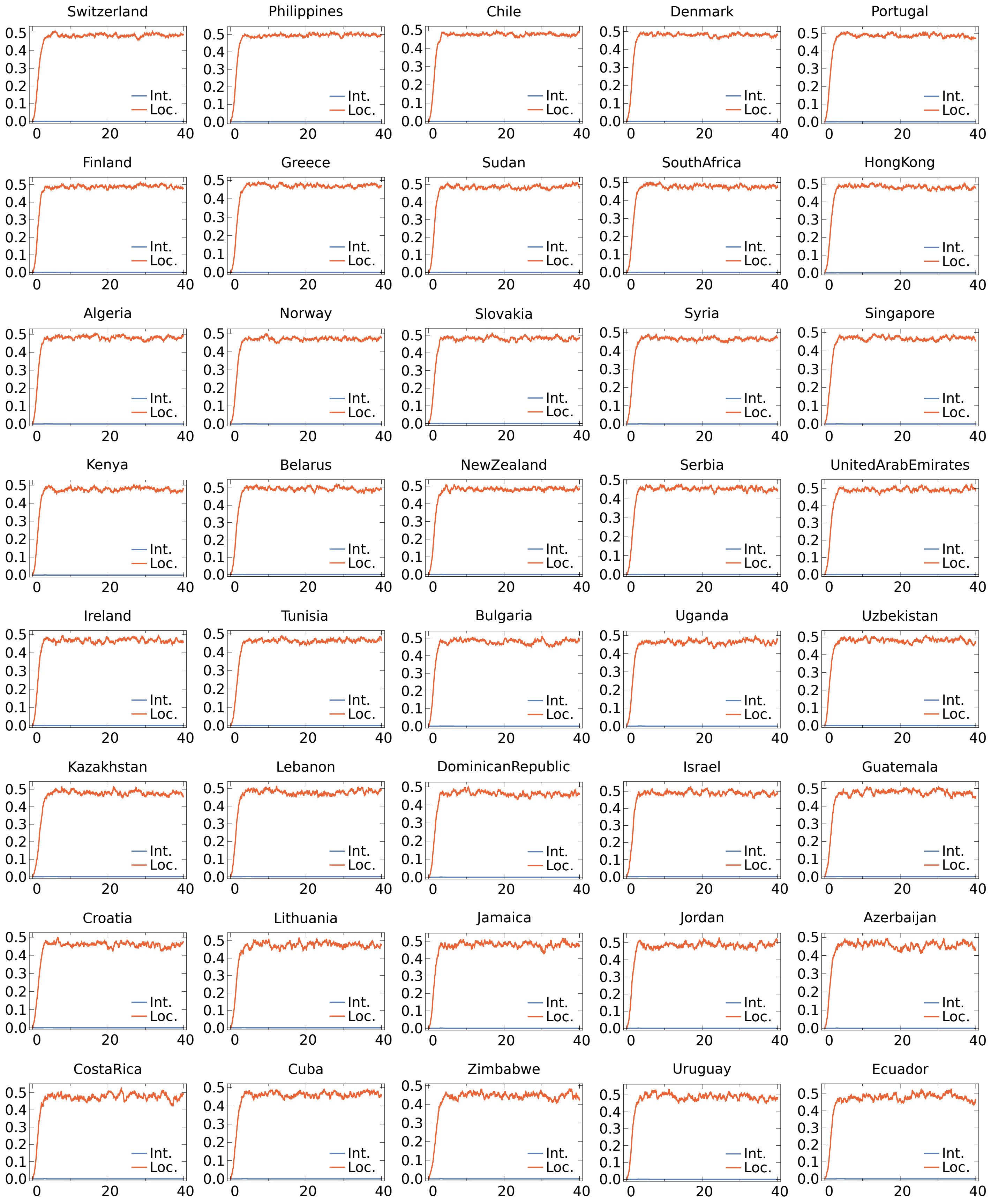

Figure S10: Evolution of network activity for the second 40 countries (continuation from Fig. S9).

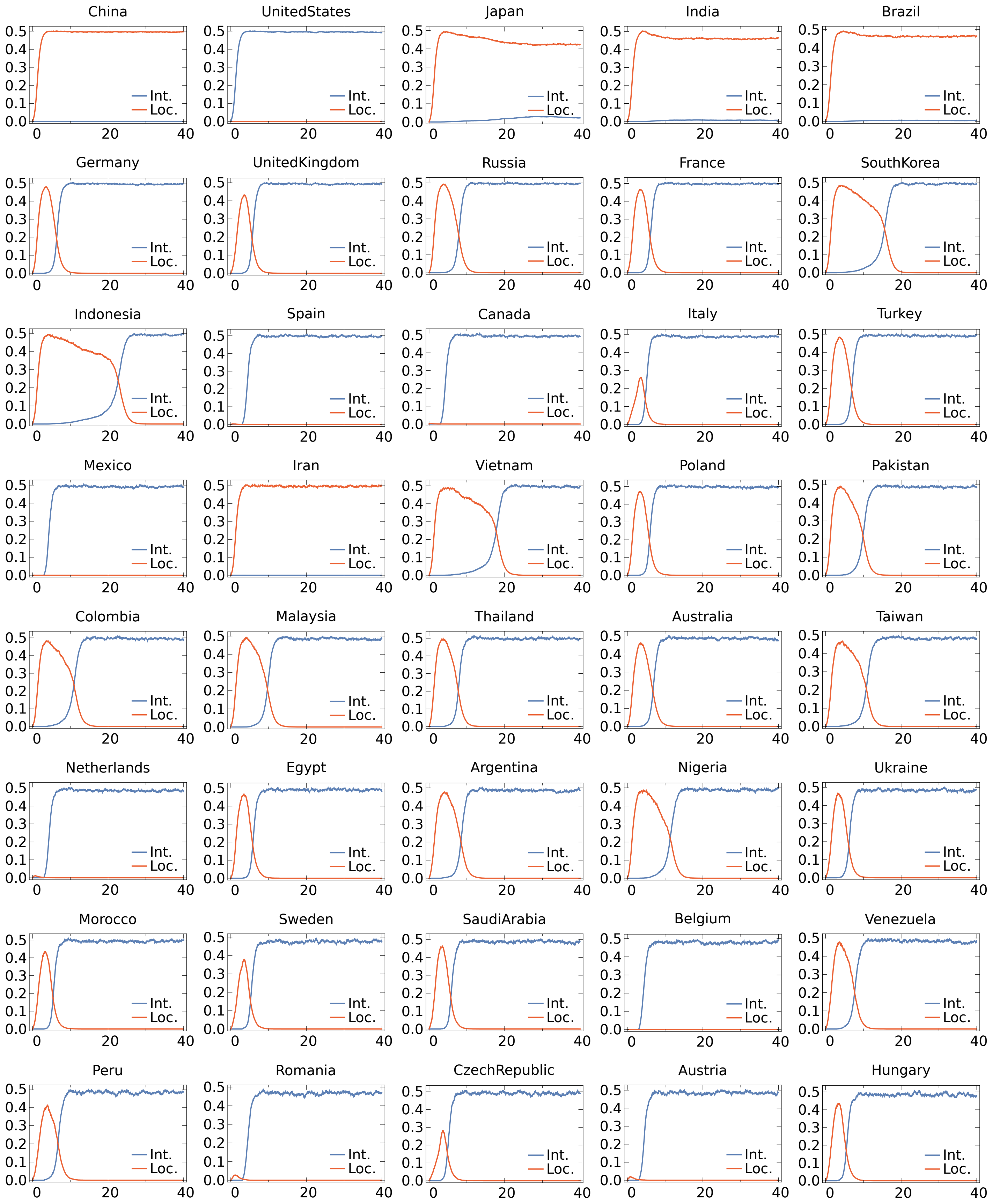

Figure S11: Evolution of network activity for the first 40 countries. Here, $\sigma = 1.5$, $\Delta t = 3$, $\alpha = 2$, and $\lambda = 0.2$ per country.

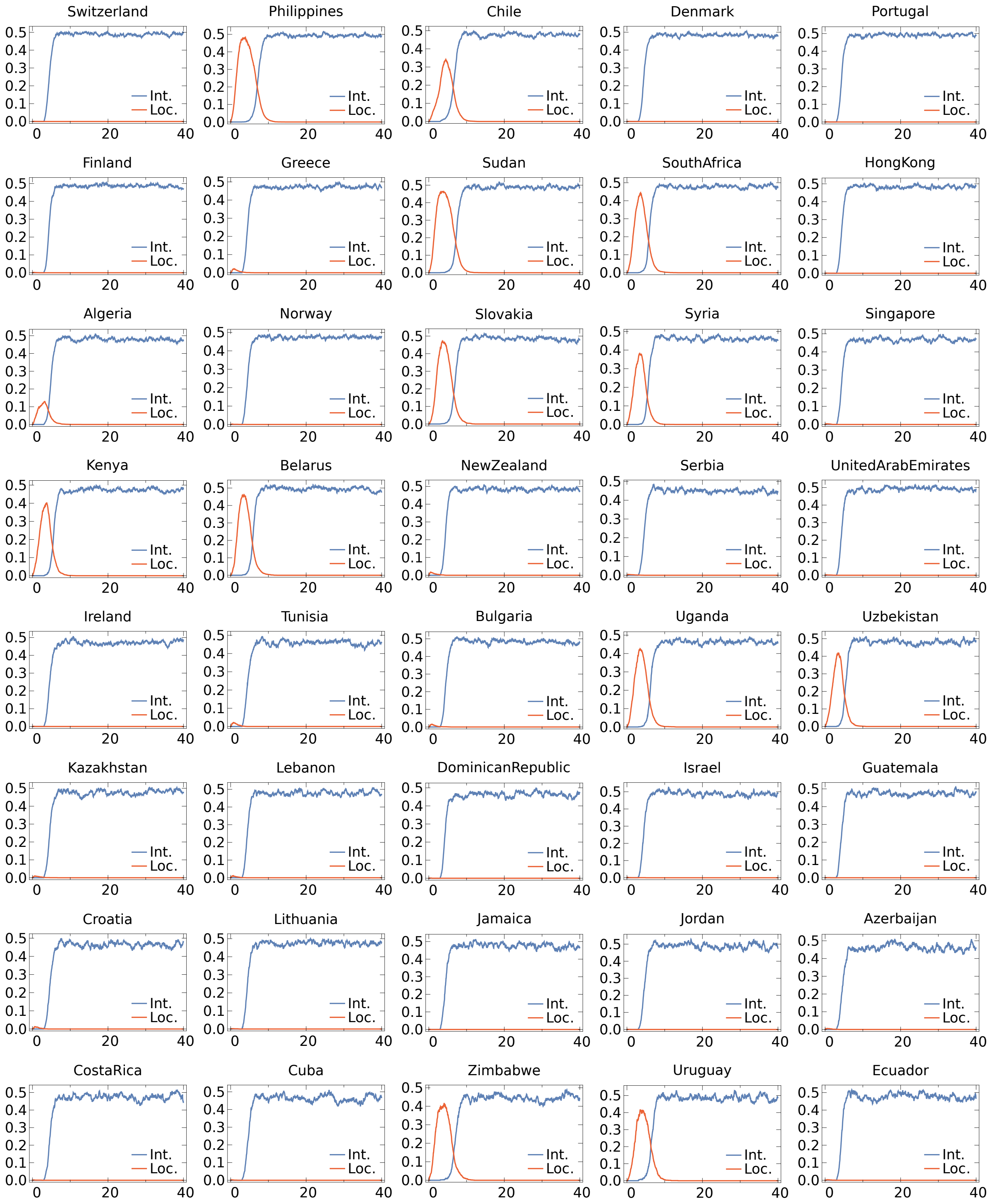

Figure S12: Evolution of network activity for the second 40 countries (continuation from Fig. S11).
